# Human urine-derived renal epithelial cells provide insights into kidney-specific alternate splicing variants

**DOI:** 10.1038/s41431-018-0212-5

**Published:** 2018-07-12

**Authors:** Elisa Molinari, Eva Decker, Holly Mabillard, James Tellez, Shalabh Srivastava, Shreya Raman, Katrina Wood, Caroline Kempf, Sumaya Alkanderi, Simon A. Ramsbottom, Colin G. Miles, Colin A. Johnson, Friedhelm Hildebrandt, Carsten Bergmann, John A. Sayer

**Affiliations:** 1 Institute of Genetic Medicine, Newcastle University, Central Parkway, Newcastle, NE1 3BZ UK; 2 Center for Human Genetics, Bioscientia, Ingelheim, Germany; 3 Renal Services, Newcastle upon Tyne Hospitals NHS Foundation Trust, Newcastle, NE7 7DN UK; 4 Northern Genetic Service, Newcastle upon Tyne NHS Foundation Trust, Newcastle, NE1 3BZ UK; 5 Royal Victoria Infirmary, Newcastle upon Tyne Hospitals NHS Foundation Trust, Newcastle, UK; 6 Department of Pediatric Nephrology, Charité University Hospital, Berlin, Germany; 7 Faculty of Medicine & Health, Leeds Institute of Biomedical and Clinical Sciences, University of Leeds, Leeds, LS9 7TF UK; 8 Department of Medicine, Boston Children’s Hospital, Harvard Medical School, Boston, MA USA; 9 Department of Medicine, University Hospital Freiburg, Freiburg, Germany

## Abstract

The majority of multi-exon genes undergo alternative splicing to produce different mRNA transcripts and this may occur in a tissue-specific manner. Assessment of mRNA transcripts isolated from blood samples may sometimes be unhelpful in determining the affect on function of putative splice-site variants affecting kidney-specific mRNA transcripts. Here we present data demonstrating the power of using human urine-derived renal epithelial cells (hUREC) as a source of kidney RNA. We report clinical and molecular genetic data from three affected cases from two families all with end-stage renal disease by 15 years of age. In both families, heterozygous variants which are predicted to effect function in *NPHP3* were found on one allele, in combination with a synonymous SNV (c.2154C>T; p.Phe718=), 18 base pairs from the exon–intron boundary within exon 15 of *NPHP3*. The only mRNA transcript amplified from wild-type whole blood showed complete splicing out of exon 15. Urine samples obtained from control subjects and the father of family 2, who carried the synonymous SNV variant, were therefore used to culture hUREC and allowed us to obtain kidney-specific mRNA. Control kidney mRNA showed retention of exon 15, while the mRNA from the patient’s father confirmed evidence of a heterozygous alternate splicing of exon 15 of *NPHP3*. Analysis of RNA derived from hUREC allows for a comparison of kidney-specific and whole-blood RNA transcripts and for assessment of the effect on function of putative splice variants leading to end-stage kidney disease.

## Introduction

Next-generation sequencing techniques, including whole-exome sequencing (WES), has seen a huge surge in gene discovery, exemplified by gene discovery in various forms of inherited renal diseases. Indeed, ciliopathy exome capture and WES approaches have been successful in identifying numerous new genetic forms of nephronophthisis, an autosomal recessive renal ciliopathy [1–3]. Similarly, isolation of whole-blood RNA followed by RT-PCR strategies for identifying variants in sets of large genes underlying certain phenotypes has been successful [4]. Routine genetic diagnostics typically verify splice-site variants identified by genetic and genomic approaches using whole-blood or fibroblast RNA to assess for aberrant splicing. This has the advantage of assessing RNA directly from the patient (rather than using in silico tools) but the disadvantage is that RNA from affected tissues is not usually available and the gene of interest may not be expressed in the available tissue. For example, assessment of mRNA transcripts isolated from blood samples may be unhelpful in determining the functional effect of putative splice-site variants affecting kidney-specific mRNA transcripts. In the modern post-genome sequencing era, interpretation and functional validation of genomic variants has become vital in the understanding of disease. Genetic variants may have consequences on RNA processing, including exon skipping, intron inclusion, and cryptic splicing. The majority of multi-exon genes undergo alternative splicing to produce different mRNA transcripts and this occurs in a tissue-specific manner [5]. Efforts to understand tissue-specific RNA transcripts have been made [6], which will hopefully increase our understanding of genotype/phenotype correlation in inherited diseases and allow the verification of the consequences of genomic variants in appropriate tissues.

Nephronophthisis is a chronic interstitial nephropathy and an autosomal recessive form of childhood kidney failure. It has numerous genetic causes, although the underlying molecular genetic change remains elusive in around 50% of cases, due to either unknown genetic causes or hidden variants in known genes. A molecular genetic diagnosis is important to understand genotype/phenotype correlations, prevent the need for a renal biopsy, and to perform screening of at risk individuals.

Here we present data demonstrating the power of using human urine-derived renal epithelial cells (hUREC) as a liquid biopsy of the kidney in order to isolate tissue-specific RNA, to demonstrate the functional effect  of a genomic DNA variant in *NPHP3* found in families with previously undiagnosed end-stage renal disease (ESRD).

## Methods

All patients consented to this study. Ethical approval was obtained from the UK National Research Ethics Service Committee Northern and Yorkshire (09/H0903/36). Following informed and written consent, blood samples were obtained from affected patients and their relatives and healthy gender and age-matched controls. WES was performed as previously described [7] and targeted sequence analysis was performed using a customized sequence capture library [8]. hURECs were isolated from urine collected from the father of family 2 and healthy age-matched donors and cultured as previously described [9, 10]. Gene variants have been submitted to www.LOVD.nl/NPHP3 (patient IDs 00164069, 00164068, and 00164805). Full details of methodology are provided in Supplementary Data.

## Results

### Clinical features and genomic investigations of two families with unexplained ESRD

In the first family, a 14-year-old presented chronic kidney disease stage 4 which progressed to unexplained ESRD by 15 years of age (Table 1). The parents were non-consanguineous and white European. There was no family history of renal disease. Renal ultrasound showed increased echogenicity and loss of cortico-medullary differentiation and a renal biopsy showed chronic interstitial fibrosis and tubular atrophy suggestive of nephronophthisis (Table 1).

**Table 1:** Clinical and molecular details of patients

ID	Consanguinity	Origin	Nucleotide^a^	Amino acid	Age at diagnosis	Symptoms at diagnosis	Renal USS	Renal histology	ESRD	Hypertension	Liver	Other extra renal symptoms
Family 1	No	German	c.3003del; c.2154C>T	p.(Phe1001Leufs*61); p.Phe718=/p.?	14 years	Metabolic acidosis, anemia, secondary hyperparathyroidism, muscle weakness	Normal-sized kidneys, reduced cortico-medullary differentiation, and increased echogenicity	Severe chronic tubulo-interstitial damage with atrophy of tubules, interstitial fibrosis and moderate interstitial inflammation	15 years	Yes, aged 14 years	No signs of liver manifestation	Left ventricular hypertrophy
Family 2 Sibling 1	No	UK	c.2694-2_2694-1delAG; c.2154C>T	p.?; p.Phe718=	6 years	Abdominal pain	Increased echogenicity	Mesangial hypercelularity, tubular atrophy, patchy interstitial fibrosis	13 years	Yes, aged 6 years	Micronodular sclerosis	Neonatal jaundice, splenomegaly
Family 2 Sibling 2	No	UK	c.2694-2_2694-1delAG; c.2154C>T	p.?; p.Phe718=	9 years	Abdominal pain and tiredness, polyuria and polydipisa	Increased echogenicity	Mesangial hypercelularity, tubular atrophy, patchy interstitial fibrosis	15 years	Yes, aged 13 years	Hepatomeagly, coarse echo texture with increased periportal echogenicity in keeping with periportal fibrosis	Splenomegaly

^a^Nucleotide reference sequence: NM_153240.3, genomic reference sequence NG_008130.1

In the second family, two siblings presented with congenital hepatic fibrosis and unexplained ESRD. The first sibling received a combined kidney and liver transplant aged 15 years (Table 1). A renal biopsy had demonstrated glomerular sclerosis and interstitial fibrosis with features suggestive of nephronophthisis (Supplementary Fig. S1). The second sibling reached ESRD at the age of 13 years and had received a renal transplant followed by a liver transplant 1 year later. The family was white British and non-consanguineous and had an unaffected male sibling (Table 1).

**Fig. 1:**
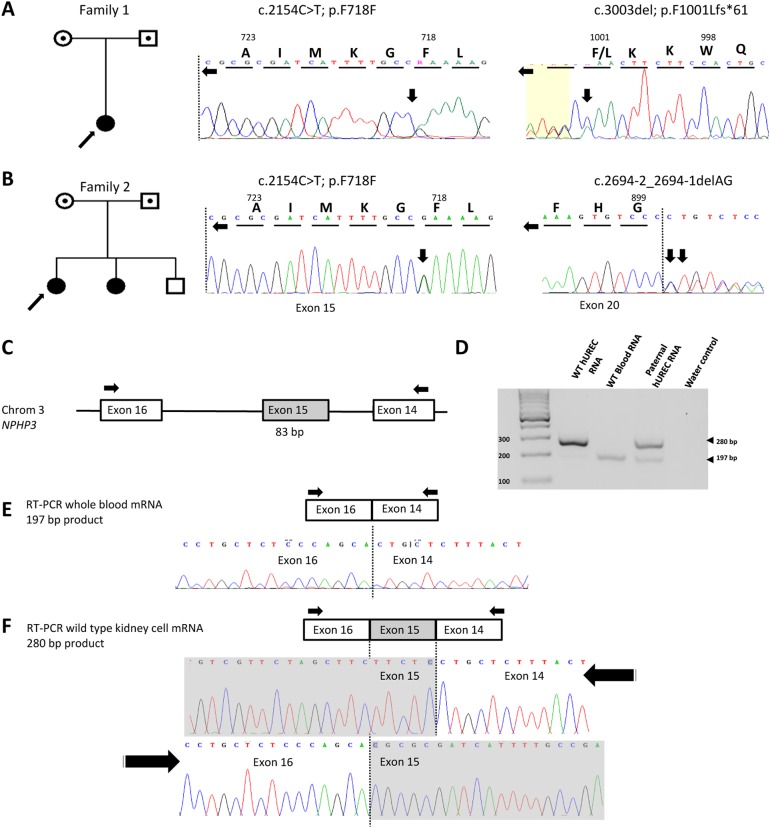
Molecular genetic investigation and tissue-specific RT-PCR reveals a functional effect of a synonymous *NPHP3* allele. **a**, **b** Pedigree diagrams (males squares, females circles, probands arrowed) and sequence chromatograms showing biallelic variants in NPHP3 in affected patient in **a** Family 1 and **b** Family 2. **c** Genomic map and RT-PCR strategy to detect abnormal splicing of exon 15 of *NPHP3*, with primers arrowed. Exons are numbered as in Olbrich et al. [12]. **d** RT-PCR using RNA isolated from wild-type (WT) hUREC, WT Whole blood and family 2 paternal hUREC. Note paternal RNA is compound heterozygous for alternate splicing of exon 15 of *NPHP3* (NM_153240.3: c.2154C>T; r.2154c>u). **e** Sanger sequencing confirms alternate splicing of *NPHP3* exon 15 in wild-type whole-blood RNA. **f** Sanger sequencing confirms inclusion of exon 15 in RNA derived from wild-type hUREC

In Family 1, targeted exon capture and sequencing of a ciliopathy panel of 121 genes, as previously reported [8, 11], identified a variant predicted to affect function in *NPHP3* (NM_153240.3, c.3003del; p.(Phe1001Leufs*61)) in combination with a synonymous SNV (c.2154C>T; p.Phe718=). The variants were present in compound heterozygous state with the frameshift variant inherited maternally and the synonymous change inherited paternally (Table 2, Fig. 1).

**Table 2:** In silico analysis of NPHP3 variants

NPHP3 variant^a^	MutationTaster	Human Splicing Finder	ExAC	gnomAD
c.3003del; p.(Phe1001Leufs*61)	Disease causing, likely NMD	n/a	Not in ExAC	Not in gnomAD
c.2694-2_2694-1delAG; p.?;	Disease causing—splice acceptor variant (HGMD CD082161; rs751527253)	Acceptor site loss	43 alleles in 121,030 alleles	77 alleles in 277,098 alleles
c.2154C>T; p.Phe718=	Disease causing (rs558637226)	^#^Possible branch point motif broken	1 allele in 121,283 alleles	3 alleles in 277,341 alleles

*NMD* nonsense-mediated decay

^a^Nucleotide reference sequence: NM_153240.3, genomic reference sequence NG_008130.1 ^#^ESEfinder, predicts an exon splicing enhancer (ESE) site is abolished thus inhibiting the binding of splicing enhancer proteins leading to aberrant splicing

In family 2, following exclusion of *PKHD1* variants by direct sequencing, WES revealed a single heterozygous variant predicted to affect function (c.2694-2_2694-1delAG) in *NPHP3*, segregating from the mother, combined with the above-mentioned synonymous SNV in *NPHP3* (c.2154C>T; p.Phe718=), segregating from the father (Table 2). The functional effect of the c.2694-2_2694-1delAG variant in leading to abnormal splicing was confirmed using RT-PCR from whole-blood RNA in the affected siblings of family 2 (Supplementary Fig. S2). In both families, the rare synonymous SNV in *NPHP3* (Table 2) was initially assumed to be benign and has been reported within ClinVar as “likely benign” (SCV000316262.1). However, given this was identified in three individuals from two different families with overlapping clinical phenotypes and the fact that no other known ciliopathy gene variants affecting function had been identified using NGS approaches in these families, additional investigations were undertaken.

The *NPHP3* c.2154C>T variant is near an exon–intron boundary (exon 15–intron 15), and therefore may have been implicated in abnormal splicing of exon 15 of *NPHP3*. In silico tools, including Human Splicing Finder suggested a functional effect, with probable disruption of exonic splicing motifs (Table 2).

mRNA isolated from whole blood from the affected patients from family 2 and unaffected healthy controls all showed complete splicing out of exon 15 of *NPHP3*, which was confirmed by Sanger sequencing, suggesting RNA splicing of this exon was tissue specific (Fig. 1). Both affected patients in family 2 had received a renal transplant, therefore RNA isolated from their urine would represent the donor. Instead, urine samples obtained from the father in family 2 and healthy control subjects were used to culture hUREC, allowing us to obtain kidney-specific mRNA. Using these tissue-derived mRNA samples, RT-PCR across exon 15 of *NPHP3* in control kidney mRNA showed retention of exon 15, confirmed by Sanger sequencing, while the RNA from the father from family 2 showed heterozygous alternative splicing in *NPHP3*, confirming a kidney-specific loss of function of the c.2154C>T allele.

## Discussion

Variants that affect function in *NPHP3* can lead to a variety of clinical presentations, as emphasized by the two families reported here. Although reported initially as a cause of adolescent NPHP [12], variants that affect function in *NPHP3* are a prominent cause of infantile NPHP and may present antenatally with enlarged cystic kidneys [13] and sometimes cause the perinatally lethal Meckel Syndrome [14]. The wide phenotypic variability, especially in extra-renal manifestations may sometimes be explained by the predicted functional effect of the variant alleles [14]. A *NPHP3* variant predicted to influence splicing may have variable effects, depending on the transcript generated and any tissue-specific effects on splicing. The fact that all three affected patients we report here reached ESRD from 13–15 years of age is typical of NPHP. The variable extra renal phenotypes, including liver fibrosis, is also typical.

The original description of *NPHP3* variants that effect function, leading to nephronophthisis in man, noted alternate mRNA transcripts of NPHP3 were expressed, with variation in splicing of exons 3b, 13, and 15 [12]. For example, the AL832877 transcript of *NPHP3* is differentially spliced, omitting exon 15, and was cloned from human lymph node, whereas the clone CA396561, derived from human retinal pigment epithelium, includes exon 15. The full-length cDNA of *NPHP3* encodes for a 1330 amino acid protein. Here we have shown that in white blood cell RNA, there is alternate splicing of exon 15 of *NPHP3*. The consequences of loss of exon 15, which has 83 nucleotides, is predicted to lead to a change in reading frame of the downstream exons of *NPHP3* and a truncated protein of just 704 amino acids. The precise function of this predicted alternate transcript is unknown, but the transcript may be subject to nonsense-mediated decay.

This report suggests that synonymous changes in known disease causing genes (such as the known NPHP genes) cannot automatically be filtered out from WES datasets before an in silico assessment of influence on splicing has been determined. The significance of synonymous variants in *PKD1*, one of the genes underlying autosomal dominant polycystic kidney disease, has recently been described [15]. Here two synonymous variants in *PKD1* were shown to induce pre-mRNA splicing defects [15]. In the case of genetically unsolved recessive cystic kidney diseases such as nephronophthisis, the finding of one variant which affects function, such as a heterozygous nonsense variant in a known genetic cause of the disease (e.g. *NPHP1*), should prompt a review of synonymous variants within the same gene to see if a synonymous variant may be the second allele which affects function.

## Conclusion

In conclusion, we confirm the functional effect of a synonymous single-nucleotide polymorphism in *NPHP3* by determining alternate splicing in kidney RNA isolated from urine-derived renal epithelial cells. This system will allow for a comprehensive comparison of kidney-specific and whole-blood RNA transcripts and for the ex vivo assessment of the functional effect of putative splice variants.

## Electronic supplementary material

Supplemental Methods and Figures
