# Supplementary material for: Human urine-derived renal epithelial cells provide insights into kidney-specific alternate splicing variants
Source: Eur J Hum Genet. 2018 Jul 12;26(12):1791–6. doi: 10.1038/s41431-018-0212-5 (PMC6244279; doi:10.1038/s41431-018-0212-5)
Supplement: Supplementary file 1 — Supplemental Methods and Figures [file 41431_2018_212_MOESM1_ESM.docx]

**Investigation of inherited renal disease: Human Urine Derived Renal Epithelial cells provide insights into kidney specific alternate splicing mutations in *NPHP3***

Elisa Molinari, Eva Decker, Holly Mabillard, James Tellez, Shalabh Srivsatava, Shreya Raman, Katrina Wood, Caroline Kempf, Sumaya Alkanderi, Simon A. Ramsbottom, Colin Miles, Colin Johnson, Friedhelm Hildebrandt, Carsten Bergmann and John A. Sayer

**Supplemental methods**

**Whole exome sequence analysis**

In brief, genomic DNA was isolated from blood lymphocytes and subjected to exome capture using Agilent SureSelect™ human exome capture arrays (Life technologies™) followed by next generation sequencing on the Illumina HiSeq™ sequencing platform. Sequence reads were mapped to the human reference genome assembly (hg19) using CLC Genomics Workbench™ (version 6.5.2) software (CLC bio, Aarhus, Denmark). Following alignment to the human reference genome, variants were filtered as previously described ([Daga, Majmundar et al. 2018](#_ENREF_7)). In the first step, variants with minor allele frequencies (MAF) >1% in the dbSNP (version 142) were excluded. In step 2, variants were evaluated for mutations in known NPHP genes ([Braun and Hildebrandt 2017](#_ENREF_4)). In step 3, non-synonymous and splice variants were ranked based on their probable impact on protein sequence and function considering evolutionary conservation among orthologs across phylogeny, as well as web-based prediction programs (PolyPhen-2, SIFT and MutationTaster). In step 4, synonymous variants in NPHP3 were evaluated only, if in compound heterozygous state with a non-synonymous variant. These compound heterozygous alleles were then functionally tested. Clinician scientists and geneticists, who had knowledge of the clinical phenotypes and pedigree structure, as well as experience with exome evaluation performed mutation calling.

**Targeted sequence analysis**

In brief, we used a customized sequence capture library ([Lu, Galeano et al. 2017](#_ENREF_10)) that targeted exons and additional 35 bp of flanking intronic sequence of genes known to cause ciliopathies. Genomic DNA was fragmented, and the coding exons of the analyzed genes as well as the corresponding exon-intron boundaries were enriched using the Roche/NimbleGen sequence capture approach (NimbleGen, Madison, Wisconsin, USA). They were then amplified and sequenced simultaneously by Illumina next generation sequencing (NGS) technology using an Illumina HiSeq 1500 system. The mean target coverage was 243x with about 98.8 % of the target regions covered at least 20x. NGS data analysis was performed by mapping paired end reads (2×100 bp) from the HiSeq instrument against the hg19 human reference genome using BWA with recommended standard settings. Mapped reads were preprocessed with SAM tools and duplicate reads were marked by Picard. Finally, GATK was applied for local realignment and base quality score recalibration of mapped reads. JSI Medical Systems software (version 4.1.2, SeqNext module) was used for visualization and final assessment of single nucleotide variants (SNVs). Variants were filtered against external databases (Exome Aggregation Consortium (ExAC), 1000 Genomes, NHLBI Exome Sequencing Project (ESP), dbSNP v.144, the Human Gene Mutation Database (HGMD ® Professional 2016.4), ClinVar) and our in-house database, focusing on rare variants with a minor allele frequency (MAF) of 1% or less. Nonsense, frameshift and canonical splice site variants were primarily considered likely pathogenic. Assessment of the damaging nature of identified missense variants was performed using bioinformatic prediction programmes like Mutation Taster, Polyphen-2, MutationAssessor, and FATHMM. *In silico* analysis of splice site effects was performed by programmes like NNSPLICE, NetGene2, Human Splicing Finder, Mutation Taster and ESE-Finder. Rare variants identified in the patient were classified according to ACMG guidelines. Mapping and coverage statistics were generated from the mapping output files using GATK. The resulting sequence data were compared to the reference sequence of the RefSeq database. High coverage enabled copy number variation analysis. Potential copy number alterations were initially identified by VarScan on mapped reads. Thereby coverage of every target region of the sample was internally normalized and compared to normalized control data of other samples of the same run by VarScan copy number mode and standard settings.

Sanger sequencing was performed to confirm segregation of *NPHP3* variants in parents and unaffected siblings where available.

**RNA preparation and RT-PCR**

RNA from human urine-derived renal epithelial cells (hUREC) and blood samples was isolated using RNeasy mini kit (Qiagen) according to the manufacturer’s instructions and quantified using a NanoDrop 2000 spectrophotometer. 1ug RNA was reverse-transcribed using an oligo-dT primer and SuperScript III Reverse Transcriptase (Thermo Fisher Scientific). The resulting cDNA was diluted 1 in 10 and used in PCR reactions. Oligonucleotide primers used for *NPHP3* PCR were forward 5’- tgtagaaacatgccctccag-3’ and reverse 5’-tgaaagagtatcttgacactggaaa-3’.

**Supplemental Figure S1**

**
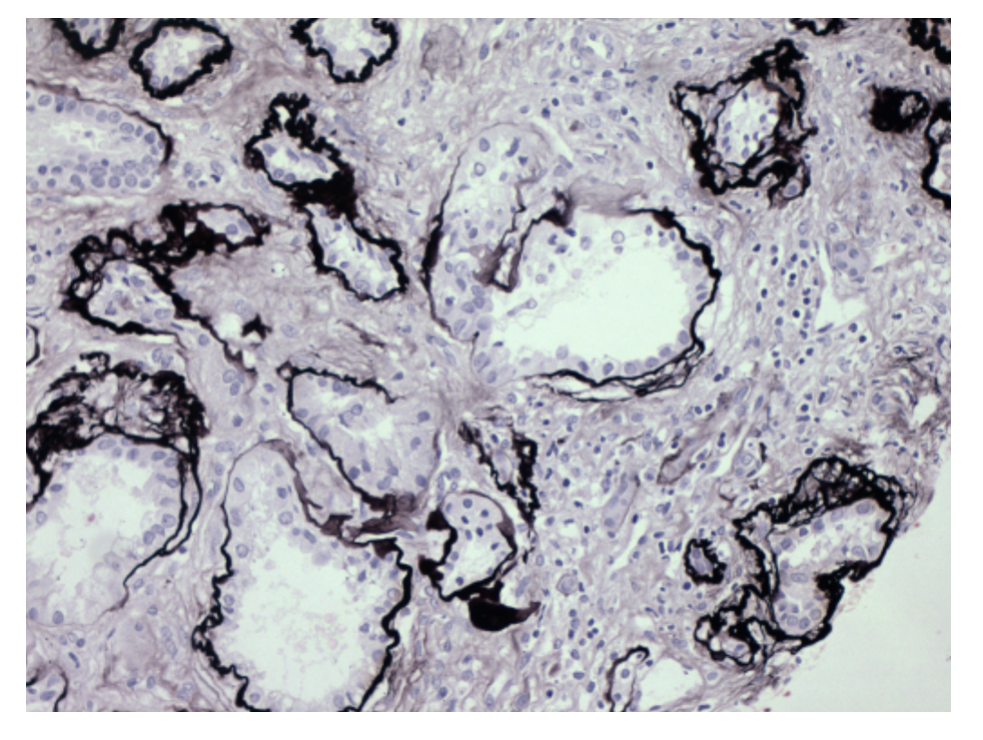
**

**Figure S1. Renal biopsy suggestive of nephronophthisis**

Renal biopsy findings from Family 2, sibling 1 shows atrophic and dilated tubules with variable wrinkling, splitting and thickening of tubular basement membranes (Silver stain x200).


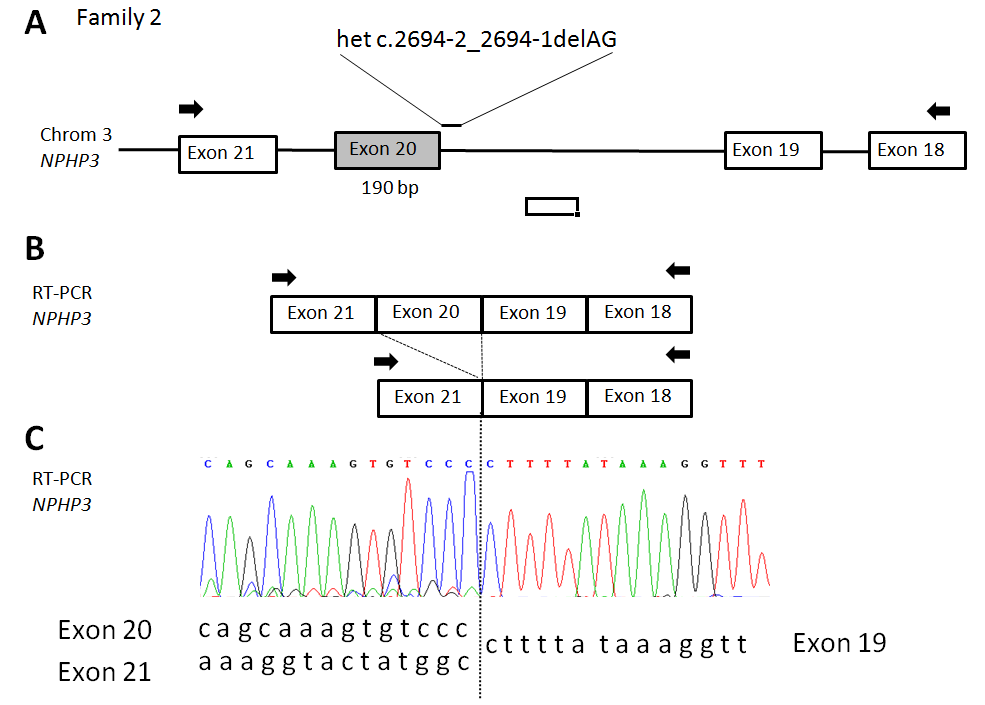


**Figure S2. RT-PCR using whole blood RNA confirms pathogenicity of splice site mutation in NPHP3**

A. Genomic map RT-PCR strategy to detect abnormal splicing of exon 20 of *NPHP3* with mutation marked.

B. Schematic of alternate splicing of exon 20 of NPHP3 and PCR primers arrowed

C. Sanger sequencing of RT-PCR product from affected patient in family 2 confirms heterozygous alternate splicing of NPHP3 exon 20 in whole blood RNA (NM_153240.3: c.2694-2_2694-1del; r.spl)
